# Research on Population Mobility and Sustainable Economic Growth From a Communication Perspective

**DOI:** 10.3389/fpsyg.2022.935606

**Published:** 2022-07-07

**Authors:** Yizhong Yao, Lei Liu

**Affiliations:** College of Economics and Management, Shihezi University, Shihezi, China

**Keywords:** comprehensive evaluation system, sustainable development, population flow, spatial effect regression, two-way fixed effect regression

## Abstract

As the global economy begins to recover, tremendous efforts will be needed to build back better to ensure decent, fulfilling, and secure work for all within an environmentally sustainable economy. Based on the perspective of communication science, this paper first constructed a comprehensive evaluation system of regional economic sustainable development indicators. Next, the least square regression model, spatial effect regression model, and two-way fixed effect regression model are used to analyze the panel data in 34 provinces and cities in China. This paper makes a detailed study on how population flow and agglomeration affect economic growth and sustainable economic development. The experiment result shows that: (1) the impact of population agglomeration on sustainable economic growth has an “inverted U” non-linear characteristic. (2) Population agglomeration promotes sustainable economic development by improving the urbanization rate. Furthermore, based on the VAR model, Granger causality analysis and co-integration technique are used to study the quantitative interaction between population growth rate and economic development level in China. The result indicates that (at the 5%-level significance): (1) in the short-term, the population growth rate has no significant effect on the economic development, while the economic development level has a significant effect on the population growth rate; (2) there is a significant negative correlation between population growth rate and economic development level in the long run.

## Introduction

According to the UN's World Urbanization Prospects report, the number of cities in developing countries with a population of more than 1 million has more than doubled in the last 50 years. Globally, the population of megacities has grown to 529 million and now accounts for 13% of the world's urban dwellers. Tokyo is currently the world's largest international business city with more than 37 million residents, while Shanghai has seen rapid population growth since the 1990's and now has 29 million residents. Problems such as traffic congestion, environmental pollution, and accelerated disease transmission caused by dense populations have also been gradually concerned (Marshall, 2005; Andrade et al., 2012). But in developing countries, where millions of people live without basic infrastructure, crowding and uneconomics generally outweigh the benefits of aggregation. More densely populated areas have easier access to resources, but productivity levels need to rise sufficiently otherwise living standards will remain low.

The demographic dividend is an important driving factor for China's rapid economic growth since the reform and opening up. However, with the change in population age structure, demographic dividend disappears, and the negative externalities of extensive development become more and more obvious. As the economic growth slows down, the marginal returns of labor, capital, and other factors show a decreasing trend. As a result, the stable economic growth, at the same time, should pay attention to economic, social, cultural, ecological, and other aspects of the balanced development, ease the path dependence problem, which is formed by the extensive development promoting the free flow, improve resource allocation efficiency, transformation and upgrading of industrial structure, improve the labor productivity and total factor productivity, and promoting economy to develop high quality. With the reduction of barriers to the flow of factors of production, industrial capital, human capital, and financial capital gather in regions with high economic development levels, and the resulting spillover effect and scale economy will promote regional economic development. Due to more perfect infrastructure, more employment opportunities, a favorable entrepreneurial environment, and higher overall income levels in economically developed areas, the trend of population concentration in large- and medium-sized cities are inevitable. At present, China is in a critical period of structural transformation. Reasonable population agglomeration can significantly improve the population age structure and industrial production efficiency in economically developed areas. However, the excessive agglomeration of the population will also make the crowding effect offset the benefits generated by the agglomeration effect, resulting in an economic recession in the city (Henderson, 2020). How to give full play to the rational population flow and efficient agglomeration effect, enhance the economic strength of the region, speed up the integration process, and achieve high-quality economic development? This paper deeply explores the relationship between population agglomeration and high-quality development, which is of great significance for promoting the efficient agglomeration of labor factors, promoting the coordinated development of urban economy and the regional integration development, and promoting the high-quality development of the regional economy.

## Related Literature and Theoretical Introduction

Herberle (1983) in the context of “the rural-urban migration reasons” for the first time put forward “push-pull” of the population flow theory, the first to put forward the rural population migration process of scientific theory; the theory of population flows originally thought rural-urban migration process mainly by the combination of “push” and “pull.” Based on this theory, Bogue (1959) comprehensively analyzed and summarized the different influencing factors of 12 kinds of thrust and 6 kinds of pull, and put forward a set of “push-pull” theories suitable for the rapid population growth in China. Lee (1966) clearly proposed for the first time that the mass migration of the floating population was a choice made under the main precondition of the above comprehensive analysis and comparison of the four activity factors (factors of destination and destination, intermediate obstacles, and personal factors). Lewis (1954) pointed out in his classic book Economic Development under the Condition of Unlimited Supply of Labor that the economic industrial structure of developing countries consists of the combination of the modern processing industry and traditional agricultural sector, as well as the modern agricultural sector which is higher than the traditional one.

### Theoretical Analysis of Population Agglomeration on the Negative Externalities of Economic Growth

Duranton and Puga (2015) believes that the agglomeration of the urban population will produce a crowding effect. With the continuous improvement of population density, a series of problems such as environmental pollution, traffic congestion, and rising living costs will occur in cities. The existence of the crowding effect hinders the continuous flow of population to cities. Jedwab et al. (2015) reported that natural population growth contributed 2.9% to urban growth in 10 African countries from 1950 to 2010, while migration contributed 1.8% to urban growth. Even growth facilitated by population inflows may be related to the thrust of poor regions rather than due to pull factors (Lipton, 1977; Bates, 1981; Bairoch, 1988; Barrios et al., 2006); the population is “expelled” from rural areas rather than attracted by the prospect of quality of life in urban areas. According to Fay and Opal (2000) and Bloom et al. (2008), urban disease and excessive population urbanization are typical cases of crowding effect, especially in some countries or regions in South America, such as Brazil, Argentina, and Uruguay, where the urbanization rate has exceeded 80%, but their economic growth and social development level seriously stagnated or lagged behind. The population mainly represented by slums is concentrated in congested environment. Bloom et al. (2008) compared urbanization driven by industrialization in Asia, which is thought to be likely to boost economic growth, with urbanization driven by demographic pressures and conflicts in Africa, which are more likely to adversely affect economic growth. Bala (2009) concluded that for Europe, the relationship between urban concentration and economic growth is positive, but there are growth traps in moderate urban concentration areas such as Asia and Latin America. Gardiner et al. (2011) showed that there was no clear relationship between agglomeration and regional growth, and further found that agglomeration had a negative impact on economic growth. Bosker (2007) reported that higher employment density means a lower growth rate. Sbergami (2002) showed that more equal distribution of economic activities in different regions stimulated national growth.

At present, there are many discussions on the economic benefits of agglomeration, but most of them focus on industrial agglomeration, while there are few literature on population agglomeration and economic development. Industrial and economic development is inseparable from individual creativity, and the development of labor-intensive industries and service industries is more dependent on a high-density population agglomeration. Therefore, it is not enough to study industrial agglomeration only, but also to discuss its impact on economic development from the perspective of population agglomeration. Some scholars believe that population agglomeration plays a positive role in promoting economic development. For example, Braun (1993) pointed out that the flow of labor to developed regions would reduce the population growth rate of the outflow regions, promote local economic growth, and realize sustainable economic growth in underdeveloped regions, while improving regional capital accumulation in the long term, narrowing the regional income gap (Braun, 1993). In other studies, urban construction land data were extracted from nighttime light data to more accurately measure the degree of urban population concentration and study the relationship between population concentration and urban economic growth. Some scholars believe that the impact of population agglomeration on economic development is in an “inverted U” shape. For example, Williamson (1965) believed that spatial agglomeration would promote the improvement of economic efficiency in the initial stage, but after agglomeration reached a certain threshold value, spatial agglomeration would inhibit economic growth, that is, the impact of agglomeration on economic growth was in an “inverted U” shape (Williamson, 1965). Brülhart and Sbergami (2009) also confirmed the threshold effect of spatial agglomeration on economic growth, which is consistent with Williamson's hypothesis.

**Figure 1:**
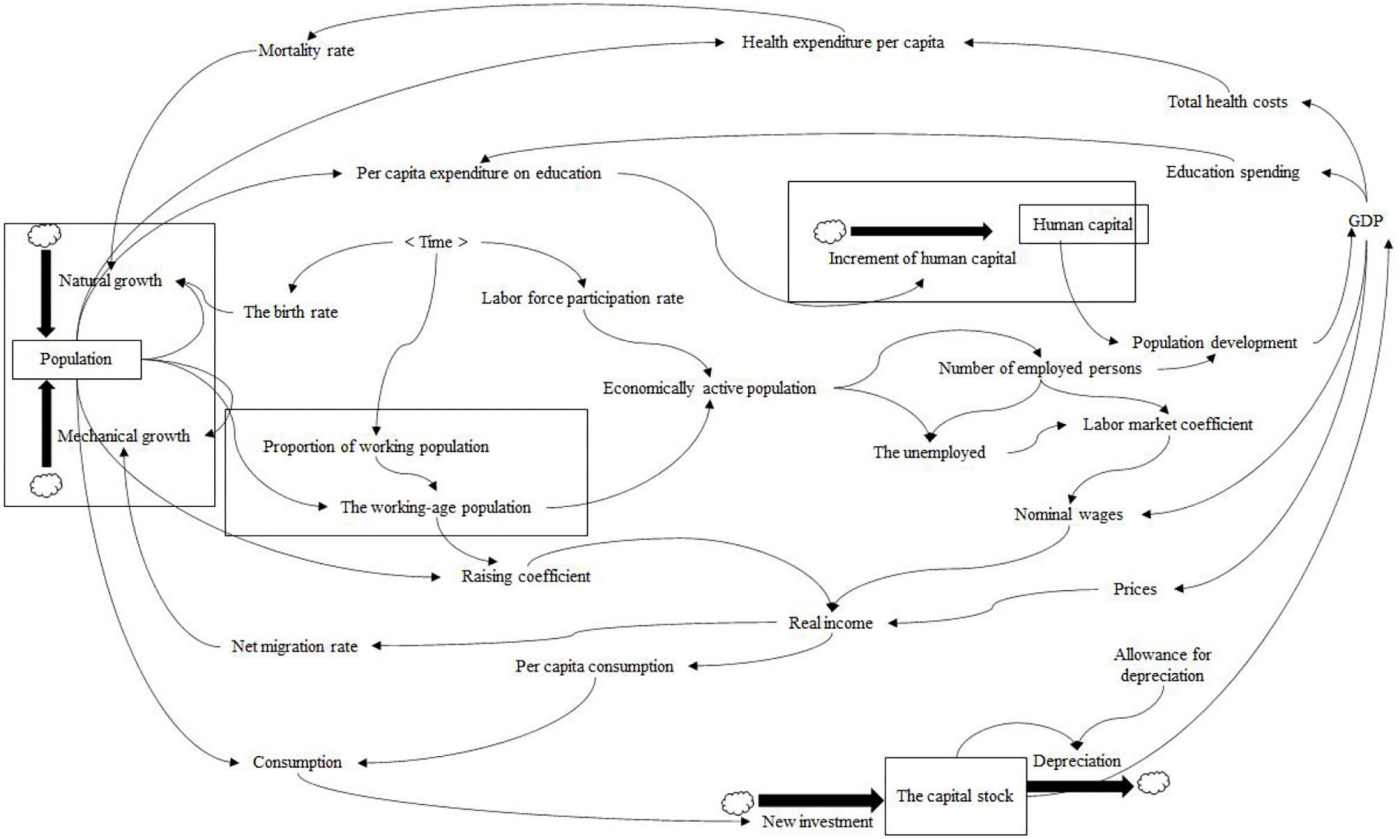
System dynamics model of population development and economic growth.

Regarding the relationship between population change and economic development, there are three main aspects: (1) Population size and economic growth. Both western economic growth theory and western classical population theory have discussed the relationship between population and economic growth in different degrees. Malthus held a pessimistic view, arguing that excessive population growth led to a vicious cycle of inadequate capital and poverty. Clark (1968) and Coale and Hoover (2015) were optimistic that population growth had a positive effect on economic growth through the increase of the labor force and the application of new knowledge and technology. Other studies (Ezeh et al., 2012) hold a moderate view that population growth is complicated by economic development. (2) Population structure and economic growth. The study holds that the effect of population structure on the macro economy is reflected in both supply and demand: one is to change the supply of the labor force by adjusting the age structure; Second, demand factors affecting aggregate consumption, savings, investment, import and export, and international capital flows (Batini et al., 2006; Bloom et al., 2015). (3) Population quality and economic growth. Research in this area mainly focuses on human capital theory and the emergence of endogenous growth theory (Romer, 1986; Lucas, 1988) promoted the research on the relationship between human capital and economic growth. Some scholars believe that human capital has a significant economic growth effect (Maitra, 2016); Others are skeptical (Vandenbussche et al., 2006). Figure 1 shows a system dynamics model of population development and economic growth.

**Figure 2:**
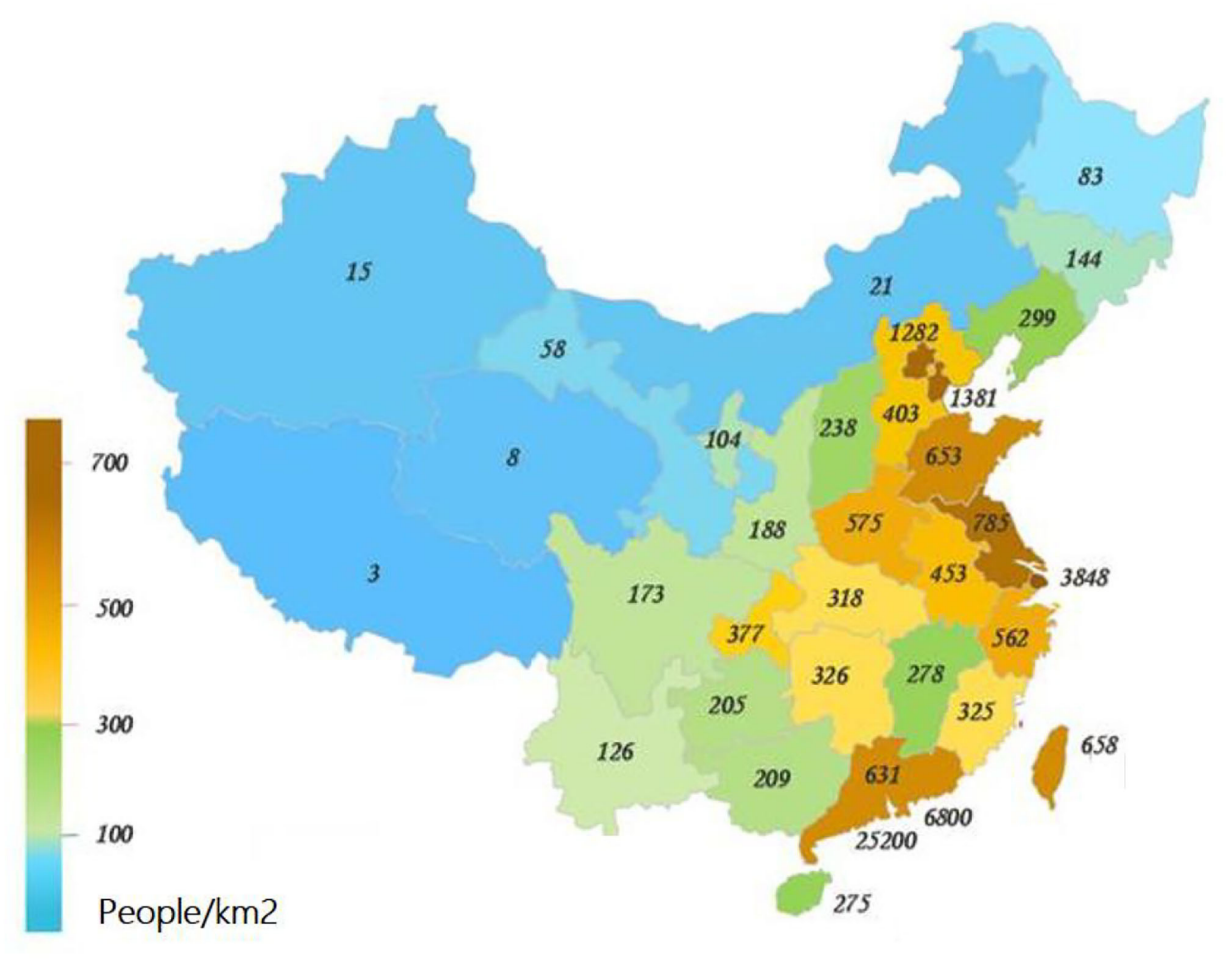
Population density of 34 provinces and cities in China in 2020.

Population development includes population quantity, population structure, and population quality. Economic growth includes two core elements of the labor force and capital. Second, the interaction mechanism flow chart between population development and economic growth should be set reasonably. On the one hand, population development affects the quantity and quality of labor force factors, and on the other hand, it influences the capital factors through consumer demand, and finally acts on economic growth. On the one hand, economic growth influences the population quantity and quality by determining the level of social security, and on the other hand, it influences the population quantity through the real income, and finally affects the population development. Finally, the internal mechanism of each core element of population development and economic growth system is determined. In the population development system, population quantity affects population structure and population quality. In the economic growth system, labor factors act on wage level, dependency burden and other variables, and change consumer demand, thus affecting capital factors. Figure 2 shows the population density of 34 provinces and cities in China in 2020.

As can be seen from Figure 2, among the 34 regions in China, the population density in the southeastern coastal region with a more developed economy is higher, which to some extent indicates that the size of the population is positively correlated with the economy.

## Research Hypothesis

The core of high-quality development is five development concepts. To study the impact of population agglomeration on high-quality development, it is necessary to analyze the economic benefits of population agglomeration from multiple perspectives. Therefore, this paper summarizes the impact of population agglomeration on innovation, green, coordination, openness, and sharing, and puts forward the impact mechanism and action mechanism of population agglomeration on high-quality development.

### Innovation

Workers with different skills gather in the same geographical space and promote the spread of knowledge and skills in the region through communication and cooperation, creating a good innovation environment and improving the innovation level of the agglomeration region (Glaeser et al., 1992; Giannetti, 2001). For enterprises, the communication externalities generated by population agglomeration promote interpersonal knowledge spillover, which is an important channel for population agglomeration to improve enterprises' innovation capability. Population agglomeration resulting from population migration to large- and medium-sized cities extends the duration of regional demographic dividend, forms human capital accumulation, and effectively improves urban labor productivity and economic efficiency. The formation of a human capital dividend will become a new driving force for economic development. However, the impact of population agglomeration on technological innovation also depends on the comprehensive effect of the urban agglomeration effect and crowding effect (Ciccone and Hall, 1996; Fan, 2005). Therefore, there may be an inverted U-shaped relationship between population agglomeration and regional innovation development.

### Coordination

Population loss will aggravate population aging and inhibit the economic growth of the destination. However, for the destination, population migration improves the local age structure, provides an abundant labor force for urbanization and industrialization, improves the local industrial agglomeration level, and reduces the economic construction cost of the destination. The improvement of population age structure and optimization of employment structure caused by population agglomeration is more conducive to regional economic growth, and the improvement of population agglomeration degree in core cities can effectively promote the upgrading of regional industrial structure and enhance regional economic competitiveness. Therefore, population agglomeration may play a positive role in promoting regional coordinated development.

### Green

When the urban population scale increases, its consumption demand and industrial scale continue to expand, idle resources are utilized more fully, an agglomeration effect is generated, and urban ecological efficiency is also improved. With the continuous increase in urban population, resources and energy required for economic activities are consumed excessively, and pollutant emissions exceed the environmental carrying capacity of cities, and ecological efficiency tends to decrease gradually. Due to the rapid development of urban industrialization and urbanization, land finance promotes the continuous rise of urban population density, urban air pollutants are far higher than the capacity of the atmospheric environment, and urban environmental air quality also decreases significantly. Therefore, there may be an inverted U-shaped relationship between population agglomeration and regional green development.

### Openness

The expansion of the population leads to an increase in the urban labor participation rate, which is conducive to the export expansion of enterprises through processing trade. In addition, population inflow is conducive to the increase of export products of local processing trade, but not conducive to the increase of export products of general trade, that is, population inflow hinders the transformation and upgrading of local enterprises from processing trade export to general trade export. Therefore, there may be an inverted U-shaped relationship between population agglomeration and regional opening and development.

### Sharing

The difference in the public service supply is one of the important factors affecting urban agglomeration; due to the unbalanced regional economic development, the economically developed areas in the public service facilities, trading into this, have the absolute advantage of employment opportunities and income, the urban population-scale improvement, and new advance of urbanization to promote the improvement of the level of regional economic development. The government's fiscal revenue increases correspondingly, and more financial resources are invested in livelihood projects, improving the level of urban education, medical care, culture, and other public services. Population agglomeration is mainly manifested by population urbanization. With the increase in the urban population, the demand for public goods and services also increases, but there is a Wagner effect in the supply of public goods related to livelihood, that is, urban public services cannot meet the rapidly growing demand in a short time (Baum and Pavan, 2012). Therefore, there may be an inverted U-shaped relationship between population agglomeration and regional shared development.

To sum up, population agglomeration plays a scale effect and knowledge spillover effect to a certain extent, thus promoting the high-quality development of local economy. However, excessive population agglomeration will produce a crowding effect and increase the cost of living and competitive pressure in cities. Because of the above analysis, this paper proposes hypothesis 1.

***H1: The impact of population agglomeration on high-quality economic development has an “inverted U-shaped” nonlinear characteristic**.*

Large-scale population agglomeration is the basis of the new urbanization. In the process of continuous population agglomeration in large- and medium-sized cities, the proportion of the urban population is increasing, urban land area is expanding at a faster pace, and economic activities are spreading outwards. As the surplus rural labor force shifts to cities, the government will relax restrictions on household registration, pay attention to the introduction of talents, and accelerate the transfer of labor population and industrial structure to the secondary and tertiary industries. The promotion of new-type urbanization is a new driving force for economic growth. The spatial agglomeration of regional population factors accelerates the urbanization process, so urbanization becomes an important way for population agglomeration to play a role in regional economic development. Population agglomeration promotes the spatial agglomeration of factors of production and economic activities and promotes the agglomeration of population and economic activities to cities through the formation of interpersonal information exchange and knowledge spillover effect through internal scale economy. Population agglomeration is extremely important for the advancement of the urbanization process, and urbanization also affects the high-quality development of the economy. Therefore, hypothesis 2 is proposed in this paper.

***H2: Population agglomeration promotes high-quality economic development*** ***by improving the urbanization rate**.*

The local government dominates the main direction of local economic development and the improvement of fiscal autonomy affects the incentive and restraint mechanism of local government. On the one hand, local governments have high access to the information within their jurisdiction, and fiscal decentralization can give play to local autonomy, improve public services, promote the development of private economy, play the role of the market and optimize resource allocation. But the fiscal decentralization through high or low is not conducive to the free movement of labor and inhibits the labor force to promote economic growth, and only under a moderate level of fiscal decentralization achievements with relatively less competition between the government, the government will pay more attention to people's livelihood and public services, attach importance to talent introduction, enhance the level of regional human capital and innovation level, and improve the quality of economic growth. On the other hand, under the performance assessment, local governments are more inclined to promote economic growth with infrastructure investment, and the fiscal expenditure of local governments has an imitation effect and a competition effect. In the short term, the construction of infrastructure by local governments can effectively stimulate local fiscal expenditure, give full play to the multiplier effect of fiscal policies, and achieve economic growth. However, in the long run, local government fiscal expenditure tends to be in the field of infrastructure, while fiscal expenditure in the fields of environmental protection, medical care, education, and science and technology is relatively reduced, which inhibits regional technological innovation to a certain extent and is not conducive to the improvement of economic growth quality. After the local government gains more financial autonomy, the economic competition between the municipal and county governments will intensify.

Furthermore, to prevent resource outflow, market segmentation and tax competition among county-level governments become more serious, hindering the improvement of resource allocation efficiency and disadvantageous to the coordinated development of regional talents and industries. Therefore, this paper proposes hypotheses 3a and 3b.

***H3a: Fiscal decentralization will enhance the promotion effect of population agglomeration on high-quality economic development;***
***H3b: Fiscal decentralization will inhibit the promotion effect of population agglomeration on high-quality economic development**.*
***H4a: Population growth is positively correlated with economic development when the population is much smaller than its environmental carrying capacity**.*
***H4b: There is a negative correlation between population growth and economic development when the population size approaches or exceeds its environmental carrying capacity***.

## Experiment Design

### Model Construction

The impact of population agglomeration on high-quality economic development may present an “inverted U” shape, promoting first and then inhibiting. To verify the above research assumptions, this paper establishes the direct influence mechanism of population agglomeration on high-quality development, and the basic model is set as follows:


(1) $$\begin{array}{l} \text{Hq}{\text{d}}_{\text{i},\text{t}}=\alpha_{0}+\alpha_{1}\text{P}{\text{a}}_{\text{i},\text{t}}+\alpha_{2}\text{P}{\text{a}}_{\text{i},\text{t}}^{2}+\alpha_{\text{c}}{\text{Z}}_{\text{i},\text{t}}+\mu_{\text{i}}+\delta_{\text{i}}+\varepsilon_{\text{i},\text{t}} \end{array}$$

In the formula, *Hqd*_*i,t*_ is the high-quality economic development level of the city *i* in period *t*; *Pa*_*i,t*_ is the population concentration degree of city *i* in period *t*; *Z*_*i,t*_ is a series of control variables represented by city *i* in period *t*; μ_*i*_ and δ represent individual and year fixed effects, respectively. ε_*i,t*_ is the random disturbance term.

Considering that the impact of population agglomeration on regional high-quality economic development may produce spatial spillover effects, a spatial panel econometric model is constructed:


(2) $$\begin{array}{l} \text{Hq}{\text{d}}_{\text{i},\text{t}}=\alpha_{0}+\rho \text{WHq}{\text{d}}_{\text{i},\text{t}}+\theta_{1}\text{WP}{\text{a}}_{\text{i},\text{t}}+\theta_{2}\text{WP}{\text{a}}_{\text{i},\text{t}}^{2}+\theta_{\text{c}}\text{W}{\text{Z}}_{\text{i},\text{t}}\\ \;+\alpha_{1}\text{P}{\text{a}}_{\text{i},\text{t}}+\alpha_{2}\text{P}{\text{a}}_{\text{i},\text{t}}^{2}+\alpha_{\text{c}}{\text{Z}}_{\text{i},\text{t}}+\mu_{\text{i}}+\delta_{\text{i}}+\varepsilon_{\text{i},\text{t}} \end{array}$$

In this formula, ρ represents the spatial autoregressive coefficient; *W* is the spatial weight matrix, and the adjacency matrix is used for spatial econometric model regression. α_0_ is the intercept term that does not change with individual characteristics. α_*i*_ is the estimated coefficient of each explanatory variable; θ_*i*_ is the spatial interaction coefficient of the explained variable. Formula (2) contains the spatial interaction term of the explanatory variable and explained variable, which is the spatial Durbin model (SDM).

In addition to the direct effect, to investigate the possible mechanism of population agglomeration on high-quality economic development, this paper empirically tests whether urbanization is an intermediary variable between the two. The specific steps are as follows: First, the coefficient α1 of population agglomeration Pa on Hqd of high-quality development in the model (1) is tested. If α1 passes the significance test, the linear regression equation of Pa on urbanization city and the regression equation of Pa and city on Hqd are constructed, respectively. The existence of mediating effect was judged by the significance of regression coefficients β1, γ1, and γ3. In addition, the mediation effect is investigated under the weight matrix of adjacent space. The specific form of the mediation effect regression model is set as follows:


(3) $$\begin{array}{l} \text{Cit}{\text{y}}_{\text{i},\text{t}}=\beta_{0}+\beta_{1}\text{P}{\text{a}}_{\text{i},\text{t}}+\beta_{2}\text{P}{\text{a}}_{\text{i},\text{t}}^{2}+\beta_{\text{c}}{\text{Z}}_{\text{i},\text{t}}+\mu_{\text{i}}+\delta_{\text{i}}+\varepsilon_{\text{i},\text{t}} \end{array}$$

(4) $$\begin{array}{l} \text{Hq}{\text{d}}_{\text{i},\text{t}}=\gamma_{0}+\gamma_{1}\text{P}{\text{a}}_{\text{i},\text{t}}+\gamma_{2}\text{P}{\text{a}}_{\text{i},\text{t}}^{2}+\gamma_{3}\text{Cit}{\text{y}}_{\text{i},\text{t}}+\gamma_{\text{c}}{\text{Z}}_{\text{i},\text{t}}+\mu_{\text{i}}+\delta_{\text{i}}\\ \;+\varepsilon_{\text{i},\text{t}} \end{array}$$

(5) $$\begin{array}{l} \text{Cit}{\text{y}}_{\text{i},\text{t}}=\beta_{0}+\rho \text{WCit}{\text{y}}_{\text{i},\text{t}}+\phi_{1}\text{WP}{\text{a}}_{\text{i},\text{t}}+\phi_{2}\text{WP}{\text{a}}_{\text{i},\text{t}}^{2}+\phi_{\text{c}}\text{W}{\text{Z}}_{\text{i},\text{t}}\\ \;+\beta_{1}\text{P}{\text{a}}_{\text{i},\text{t}}+\beta_{2}\text{P}{\text{a}}_{\text{i},\text{t}}^{2}+\beta_{\text{c}}{\text{Z}}_{\text{i},\text{t}}+\mu_{\text{i}}+\delta_{\text{i}}+\varepsilon_{\text{i},\text{t}} \end{array}$$

(6) $$\begin{array}{l} \text{Hq}{\text{d}}_{\text{i},\text{t}}=\gamma_{0}+\rho \text{WHq}{\text{d}}_{\text{i},\text{t}}+\theta_{1}\text{WP}{\text{a}}_{\text{i},\text{t}}+\theta_{2}\text{WP}{\text{a}}_{\text{i},\text{t}}^{2}+\theta_{3}\text{WCit}{\text{y}}_{\text{i},\text{t}}\\ \;+\theta_{\text{c}}\text{W}{\text{Z}}_{\text{i},\text{t}}+\gamma_{1}\text{P}{\text{a}}_{\text{i},\text{t}}+\gamma_{2}\text{P}{\text{a}}_{\text{i},\text{t}}^{2}+\gamma_{3}\text{Cit}{\text{y}}_{\text{i},\text{t}}+\gamma_{\text{c}}{\text{Z}}_{\text{i},\text{t}}+\mu_{\text{i}}\\ \;+\delta_{\text{i}}+\varepsilon_{\text{i},\text{t}} \end{array}$$

In addition, to investigate whether population agglomeration may be affected by fiscal decentralization on high-quality economic development, the interaction term between population agglomeration and fiscal decentralization (*Pa*×*Fd*) is added into Equation (1), and the general fixed effect model and spatial panel effect model are used for the empirical test. The specific form is set as follows:


(7) $$\begin{array}{l} \text{Hq}{\text{d}}_{\text{i},\text{t}}={\text{λ}}_{0}+{\text{λ}}_{1}\text{P}{\text{a}}_{\text{i},\text{t}}+{\text{λ}}_{2}\text{P}{\text{a}}_{\text{i},\text{t}}^{2}+{\text{λ}}_{3}\text{P}{\text{a}}_{\text{a}}\times \text{F}{\text{d}}_{\text{i},\text{t}}+\lambda_{\text{c}}{\text{Z}}_{\text{i},\text{t}}+\mu_{\text{i}}\\ \;+\;\delta_{\text{i}}+{\text{ε}}_{\text{i},\text{t}} \end{array}$$

(8) $$\begin{array}{l} \text{Hq}{\text{d}}_{\text{i},\text{t}}=\lambda_{0}+\rho \text{WHq}{\text{d}}_{\text{i},\text{t}}+\varphi_{1}\text{WP}{\text{a}}_{\text{i},\text{t}}+\varphi_{2}\text{WP}{\text{a}}_{\text{i},\text{t}}^{2}+\varphi_{3}\text{WPa}\\ \;\times \text{F}{\text{d}}_{\text{i},\text{t}}+\varphi_{\text{c}}\text{W}{\text{Z}}_{\text{i},\text{t}}+\lambda_{1}\text{P}{\text{a}}_{\text{i},\text{t}}+\lambda_{2}\text{P}{\text{a}}_{\text{i},\text{t}}^{2}+\lambda_{3}\text{Pa}\times \text{F}{\text{d}}_{\text{i},\text{t}}\\ \;+\lambda_{\text{c}}{\text{Z}}_{\text{i},\text{t}}+\mu_{\text{i}}+\delta_{\text{i}}+\varepsilon_{\text{i},\text{t}} \end{array}$$

### Variable Measure and Description

#### Explained Variable: High-Quality Economic Development Level

Based on the connotation and influencing factors of high-quality economic development, this paper comprehensively considers the availability of urban data and constructs an evaluation index system of high-quality urban economic development from five dimensions of innovation, coordination, green, openness, and sharing, as listed in Table 1. The measurement of the total number of people employed in the whole society; Expected output is expressed as real GDP after the adjustment; Undesired output is measured by the urban-rural income ratio and inverted into the desired output. The average salary and per capita disposable income of employed workers are also selected for the corresponding deflator of the provinces where each city is located.

**Table 1:** Evaluation system of high-quality development of urban agglomeration.

**Evaluative Dimension**	**Basic indicators**	**Indictor**
Innovation	Input in scientific and technological innovation	Science and technology financial expenditure/general financial budget expenditure
	Personnel promotion investment	Expenditure on education/Number of college students
	Number of patents granted per capita	Number of three types of patents granted/resident population
Coordinate	Inclusive TFP	Inclusive TFP index
	The consumption structure	Consumer spending /GDP
	Rationalization of industrial structure	Thayer index
	Advanced industrial structure	Output value of tertiary industry/output value of secondary industry
Coordinate	Discharge of wastewater per unit of industrial added value	Industrial wastewater discharge/total industrial output value
	Exhaust gas emission per unit of industrial added value	Industrial sulfur dioxide emissions/total industrial output value
	Smoke (powder) dust emission per unit of industrial added value	Industrial smoke (powder) dust emission/total industrial output value
	Sewage treatment rate	Centralized treatment rate of sewage treatment plant
	Domestic garbage disposal rate	Harmless treatment rate of household garbage
	Afforestation coverage rate of built-up area	Afforestation coverage rate of built-up area
Open	Foreign trade	Fdi actually utilized /GDP
Shared	Number of hospital beds per capita	Number of hospital beds/resident population
	Number of doctors per capita	Number of practicing (assistant) physicians/resident population
	Income level	Average salary of employees on the job
	Per capita disposable income	Per capita disposable income

#### Core Explanatory Variable: Population Agglomeration

To fully reflect the spatial distribution of population and analyze the concentration degree of population distribution in each city, the spatial distribution of population is measured by location entropy. In this paper, the degree of population agglomeration is measured by the degree of population geographic concentration, which increases with the increase of the value. The calculation formula is:


(9) $$\begin{array}{l} Pa_{it}=\frac{Pop_{it}/Pop_{t}}{Arc_{it}/Arc_{t}} \end{array}$$

Where, *Pa*_*it*_ represents the geographic concentration of population in the region *i* in *t* year; *Pop*_*it*_ and *Arc*_*it*_ represent the total resident population and land area at the end of year *t* in region *i*, respectively. *Pop*_*t*_ and *Arc*_*t*_, respectively, represent the permanent population and land area of 40 cities in China at the end of year T.

#### Intermediary Variable: Urbanization Level (City)

The urbanization rate of the permanent urban population is expressed as the ratio of the permanent urban population to the total regional population.

#### Moderating Variable: Fiscal Decentralization Degree

It is expressed by the ratio of revenue and expenditure in the fiscal budget, that is, the ratio of revenue and expenditure in the fiscal budget.

#### Control Variables

The degree of government intervention (Gov) is expressed by the proportion of government financial expenditure in GDP; Financial development level (Fin) is expressed by the ratio of outstanding loans of financial institutions to GDP; Level of informationization (Tel) is expressed as per 10 million telephone users; The degree of openness is expressed by the proportion of total imports and exports to GDP.

### Data Sources and Statistical Description

In this paper, the data of 40 prefecture-level and above cities in 31 provinces and cities in the China region from 2008 to 2020 were taken as samples, and a total of 600 panel observations were obtained. The missing values were corrected by the interpolation method. The above data are from the Statistical yearbook of Chinese Cities, The Statistical Yearbook of China.

## Results

### Analysis of Benchmark Regression Results

Table 2 reports the estimation results of Equation (1). Equations (1–3) are listed as regression results using OLS, (4) to (6) are listed as regression results using bidirectional fixed effect (FE). The estimated coefficients of explanatory variables and control variables show consistent sign direction and significance. The goodness of fit of FE estimation method is greatly improved compared with the OLS estimation method. Specifically, the estimated coefficient of Pa, the core explanatory variable of Model 1 and Model 4, is significantly positive at a 1% confidence level. The quadratic term of Pa was added into models 2 and 5. The first term coefficient was still significantly positive at a 1% confidence level, and the quadratic term coefficient was significantly negative at a 5% confidence level. Control variables were added into models 3 and 6. The coefficient of the first term was still significantly positive at the 1% confidence level and the coefficient of the second term was significantly negative at the 1% confidence level. After the addition of quadratic terms and control variables, the goodness of fit of the model is gradually improved. The above results show that the impact of population agglomeration on high-quality economic development shows a significant “inverted U” shaped relationship, and H1 has been preliminarily verified. Therefore, the critical value can be calculated according to the estimated coefficients of the first and second terms of population agglomeration. According to Model 6, it can be obtained that the critical value is 3.278 when the population agglomeration has the greatest promoting effect on high-quality economic development. If the population agglomeration degree is lower than this value, its influence on high-quality development is displayed as the left rising stage of the “inverted U” shaped curve, that is, the increase of population agglomeration degree will promote high-quality economic development. If the degree of population agglomeration exceeds the critical value, its impact on high-quality development is shown on the right side of the inverted U-shaped curve, that is, the increase of population agglomeration will inhibit high-quality economic development. By observing the data on urban population agglomeration in China, the average population agglomeration degree is 1.153, and the population agglomeration degree of most cities is at a low level. The variation trend of population agglomeration degree of each city is different over time, and some cities show an upward trend, such as Shanghai, Nanjing, and Suzhou. Some cities remained stable, such as Zhenjiang, Huangshan, and Huzhou. Some cities showed a downward trend, such as Suqian, Quzhou, and Anqing. Of 41 cities, only Shanghai is over the inflection point, this is due to the Shanghai economy more developed, the traffic infrastructure and public service, transaction cost, and employment have the absolute advantage of business opportunities and income, and the floating population has a strong pulling force, making the population to concentrate in Shanghai and improve the population agglomeration degree of Shanghai. However, excessive population agglomeration also leads to urban congestion, resulting in high housing prices, traffic jams, rising living costs, an insufficient supply of public services, and many other social and economic problems, thus inhibiting high-quality economic development. In model 6, the degree of government intervention in each city is positively correlated with the high-quality economic development, but not significantly, indicating that the government's expansionary fiscal policy cannot effectively promote the high-quality regional economic development. The level of financial development and informatization are positively correlated with the high-quality economic development and pass the significance test of 1%, indicating that establishing an effective and perfect financial market, improving the efficiency of capital allocation, reducing market risks, and strengthening interpersonal information exchange are conducive to promoting the high-quality regional economic development. The coefficient value of openness degree is negative, but not significant, indicating that excessive reliance on international trade cannot effectively promote the improvement of regional economic development quality, and while ignoring the strong domestic demand, it is easy to form technological dependence, which is not conducive to regional innovation.

**Table 2:** Baseline regression results of population agglomeration affecting high-quality development.

**Variable**	**(1)**	**(2)**	**(3)**	**(4)**	**(5)**	**(6)**
Population agglomeration	0.031^***^ (7.4)	0.059^***^ (4.9)	0.026^***^ (3.1)	0.088^***^ (5.1)	0.119^***^ (4.7)	0.109^***^ (4.0)
Population concentration square term		−0.014^**^ (−2.64)	−0.012^***^ (−3.70)		−0.012^**^ (−1.04)	−0.022^***^ (−3.4)
Degree of government intervention			−0.02 (−0.14)			0.034 −1.5
Level of financial development			0.091^***^ (10.43)			0.018^***^ (2.41)
Level of informatization			0.076^***^ (7.31)			0.047^***^ (4.65)
Degree of openness			0.021 (1.26)			−0.051 (−2.11)
Constant term	0.241^***^ (34.6)	0.199^***^ (24.7)	0.151^***^ (9.51)	0.069^***^ (5.13)	0.048^**^ (2.331)	0.079^***^ (2.5)
*R* ^2^	0.13	0.141	0.51	0.86	0.84	0.899
F	65.41	41.81	94.82	241.14	251.54	231.41
N	600	600	600	600	600	600

*t-Value in parentheses*;

***, **, and * *represent P < 0.01, P < 0.05 and P < 0.1, respectively*.

### Analysis of Spatial Effect Regression Results

Before the validation of the spatial econometric model, this paper first tested the spatial correlation of the explained variable, namely, high-quality economic development, as listed in Table 3. Under the adjacency spatial weight matrix, the global Moran index of the high-quality development level of China from 2008 to 2020 is positive and passes the significance test at 10% level, indicating that the high-quality economic development of each region does not exist in isolation, that is, there is a significant spatial dependence.

**Table 3:** Global Moran's/index 2008 – 2020.

**Year**	**Moran value**	**Z value**	**Year**	**Moran value**	**Z value**
2008	0.33^***^	4.42	2014	0.51^***^	4.59
2008	0.41^***^	3.51	2015	0.48^***^	5.07
2009	0.32^***^	2.8	2016	0.34^***^	4.08
2010	0.25^***^	2.69	2017	0.31^***^	3.75
2011	0.15^**^	1.84	2018	0.31^***^	3.84
2012	0.13^*^	1.56	2020	0.29^***^	3.12
2013	0.23^***^	2.91	

***, **, and * *represent P < 0.01, P < 0.05 and P < 0.1, respectively*.

LM test was used for model selection in this paper. Table 4 shows that BOTH LM-LAG and LM-error are significant at a 1% confidence level, indicating that there is a spatial effect between variables, that is, OLS is biased. Moreover, the Robust LM-lag and Robust LM-error statistics are also significant at a 1% confidence level, indicating that SDM is superior to SLM and SEM. Therefore, this paper uses the Spatial Dobin model.

**Table 4:** Model test of spatial econometric model.

**Inspection**	**Statistics**	***P-*values**	**Inspection**	**Statistics**	***P*-values**
LM (error)	69.51^***^	0	Robust LM (error)	64.421^***^	0
LM (lag)	21.41^***^	0	Robust LM (lag)	12.114^***^	0

***, **, and * *represent P < 0.01, P < 0.05, and P < 0.1, respectively*.

Table 5 shows the bidirectional fixed effects of the static spatial econometric model. In order to test the robustness of the model, the estimation results of SDM, SAR and SEM models are listed in this paper. The spatial autoregressive coefficient and error coefficient are significantly positive, which indicates that there is spatial convergence and dependence between population agglomeration and high-quality economic development. When the neighboring population concentration degree is high, the local population concentration degree will also improve, and when the neighboring economic high-quality development level is high, the local economic development quality is also high. Population agglomeration in the region of the influence on the development of the economy, high-quality “inverted U” type characteristics of SDM model to calculate the inflection point of the value of 3.139, compared with common panel inflection point value of the fixed effect model was only slightly lower, but the conclusion remains valid, and the significance of control variables and influence the direction and common fixed effects model regression results are identical. The robustness of parameter estimation of each variable was enhanced, and H1 was verified.

**Table 5:** Regression results of a spatial model of population agglomeration affecting high-quality development.

**Variable**	**Regardless of spatial effects**	**Consider the spatial effect**		
		**Spatial durbin model (SDM)**	**Spatial autoregressive model (SAR)**	**Spatial error model (SEM)**
Population concentration	0.121^***^ −5.4	0.191^***^ −5.3	0.171^***^ −5.5	0.126^***^ −5.4
Population concentration square term	−0.031^***^ (−4.12)	−0.041^***^ (−5.13)	−0.041^***^ (−5.69)	−0.023^***^ (−4.11)
Degree of government intervention	0.021 (1.51)	0.014 (1.54)	0.013 (1.41)	0.031 (1.44)
Level of financial development	0.018^***^ (3.22)	0.011^***^ (3.21)	0.044^***^ (3.31)	0.031^***^ (3.3)
Level of informatization	0.057^***^ (4.61)	0.049^***^ (4.51)	0.049^***^ (4.57)	0.046^***^ (4.37)
Degree of openness	−0.013 (−1.31)	−0.012* (−1.24)	−0.014 (−1.51)	−0.014 (−1.51)
ρ/λ		0.310^***^ (3.67)	0.33^***^ (4.01)	0.39^***^ (4.1)
*R* ^2^	0.865	0.712	0.591	0.541
logL	212.12	289.21	277.34	278.84
N	600	600	600	600

***, **, and * *represent P < 0.01, P < 0.05, and P < 0.1, respectively*.

### Mediation Effect Analysis

The general panel and spatial panel models mentioned earlier have confirmed the “inverted U-shaped” impact of population agglomeration on high-quality economic development and analyzed the impact mechanism of population agglomeration on high-quality economic development from the perspective of urbanization. To verify this mechanism, the mediation effect model is used for the empirical test below, and the regression results are listed in Table 6. When not considering the spatial effect of model 1, population agglomeration in a coefficient is positive, the quadratic term coefficient is negative, at a 1% significant level, confirming the population agglomeration effect on urbanization there is “U” type, model 2 gamma estimated coefficients of 1–0.101 and a significant at 1% level, below the benchmark in the regression model to estimate coefficient alpha 1. And γ3 is significantly positive at 10% level, indicating that urbanization is the mechanism of population agglomeration on high-quality economic development. After considering the spatial effect, the above regression coefficients and significance are consistent, indicating that the conclusion of this study is robust, and H2 has been verified.

**Table 6:** Test results of the mediating mechanism of population agglomeration affecting high-quality development.

**Variable**	**Regardless of spatial effects**		**Consider the spatial effect**	
	**Urbanization**	**High quality development level**	**Urbanization**	**High quality development level**
Population concentration	0.214^***^ (9.41)	0.121^***^ (5.14)	0.233^***^ (12.31)	0.041^***^ (2.9)
Population concentration square term	−0.029^***^ (−6.34)	−0.021^***^ (−3.33)	−0.013^***^ (−7.25)	−0.009^***^ (−3.91)
Urbanization		0.054^*^ (1.51)		0.081^**^ (2.41)
Degree of government intervention	0.003 (0.22)	0.031 (1.5)	0.011 (0.41)	0.019 (1.31)
Level of financial development	−0.019^**^ (−2.14)	0.044^***^ (3.41)	−0.007 (−0.88)	0.023^***^ (2.51)
Level of informatization	−0.005 (−0.24)	0.051^***^ (4.44)	−0.021 (−0.99)	0.045^***^ (4.98)
Degree of openness	0.041^***^ (4.24)	−0.022^*^ (−1.51)	0.041^***^ (5.11)	−0.031^**^ (−2.5)
ρ			0.251^***^ (5.32)	0.292^***^ (5.41)
*R* ^2^	0.731	0.791	0.42	0.481
logL	145.31	263.13	183.113	288.54
N	600	600	600	600

***, **, and * *represent P < 0.01, P < 0.05, and P < 0.1, respectively*.

### Analysis of the Regulatory Effect

Considering that the effect of population agglomeration on high-quality economic development will be affected by fiscal decentralization, to test the interaction between population agglomeration and fiscal decentralization, the general fixed effect model and spatial panel effect model are used for empirical tests, and the regression results are listed in Table 7. Without considering the spatial effect, the coefficient of the interaction term between population agglomeration and fiscal decentralization is negative but not significant. However, after considering the spatial effect, it shows a significant negative impact, indicating that the improvement of fiscal decentralization inhibits the promotion effect of population agglomeration on the high-quality development of the local economy. Therefore, H3a is not established and H3b is verified.

**Table 7:** Test results of the moderating mechanism of population agglomeration affecting high-quality development.

**Variable**	**Regardless of spatial effects**	**Adjacency matrix**
Population agglomeration	0.153^***^ (5.41)	0.144^***^ (5.15)
Population concentration square term	−0.021^***^ (−4.41)	−0.020^***^ (−3.89)
Interaction of population agglomeration and fiscal decentralization	−0.003 (−1.41)	−0.0078^**^ (−2.31)
Degree of government intervention	0.031 (1.45)	0.034 (1.53)
Level of financial development	0.024^***^ (3.54)	0.025^***^ (3.56)
Level of informatization	0.051^***^ (4.31)	0.034^***^ (3.31)
Degree of openness	−0.031 (−1.44)	−0.015^*^ (−1.21)
*R* ^2^	0.841	0.49
logL	271.23	299.11
N	600	600

***, **, and * *represent P < 0.01, P < 0.05, and P < 0.1, respectively*.

### Robustness Test

To further ensure the robustness of the empirical results above, this paper replaced the measurement method of explained variables. The level of high-quality economic development was represented by the per capita GDP after the adjustment and logarithm, and the robustness test was carried out by using the spatial econometric model under the static panel and adjacency matrix. The regression results are listed in Table 8. No matter in the benchmark model, the mediating effect model, or the moderating effect model, the sign and significance of the estimated coefficients of the core variables did not change significantly, which further confirmed the robustness of the empirical results mentioned above.

**Table 8:** Robustness test results.

**Variable**	**Regardless of spatial effects**			**Consider the spatial effect**		
Population agglomeration	0.821^***^ (10.31)	0.431^***^ (4.91)	0.812^***^ (10.13)	0.815^***^ (10.30)	0.355^***^ (4.81)	0.898^***^ (11.41)
Population concentration square term	−0.051^***^ (−4.91)	−0.031^*^ (−1.31)	−0.061^***^ (−5.12)	−0.051^***^ (−4.91)	−0.024 (−1.31)	−0.053^***^ (−4.98)
Urbanization		1.298^***^ (10.1)			1.381^***^ (11.3)	
Interaction of population agglomeration and fiscal decentralization			−0.121^***^ (−11.41)			−0.181^***^ (-13.41)
Degree of government intervention	0.11 (1.09)	0.052 (0.52)	0.01 (0.13)	0.081 (1.41)	0.054 (0.56)	0.031 (0.43)
Level of financial development	−0.21^***^ (−9.51)	−0.191^***^ (−9.45)	−0.149^***^ (−8.91)	−0.201^***^ (−10.16)	−0.199^***^ (−9.61)	−0.151^***^ (−8.99)
Level of informatization	−0.03 (−1.1)	−0.051 (−1.62)	−0.116^***^ (−2.62)	−0.046^*^ (−1.42)	−0.03 (−1.1)	−0.142^***^ (−3.12)
Degree of openness	0.069^**^ (2.41)	−0.013 (−0.09)	0.044 (1.2)	0.078^**^ (2.81)	−0.02 (−0.91)	0.031^**^ (1.21)
ρ				0.151^**^ (2.21)	0.14^*^ (1.91)	0.241^***^ (3.54)
*R* ^2^	0.912	0.934	0.9411	0.412	0.321	0.399
logL	611.21	615.33	652.13	633.25	651.21	678.54
N	600	600	600	600	600	600

***, **, and * *represent P < 0.01, P < 0.05, and P < 0.1, respectively*.

### Empirical Analysis of VAR Model

Let D represent per capita GDP and S represent population growth rate:


(10) $$\begin{array}{l} {\text{D}}_{\text{t}}={\text{a}}_{0}+{\text{a}}_{1}^{*}{\text{D}}_{\text{t}-1}+{\text{a}}_{2}^{*}{\text{D}}_{\text{t}-2}+{\text{a}}_{3}^{*}{\text{D}}_{\text{t}-3}+\ldots +{\text{a}}_{\text{k}}^{*}{\text{D}}_{\text{t}-\text{k}}\\ \;+{\text{b}}_{1}^{*}{\text{S}}_{\text{t}-1}+{\text{b}}_{2}^{*}{\text{S}}_{\text{t}-2}+{\text{e}}_{1_{\text{t}}} \end{array}$$

(11) $$\begin{array}{l} {\text{S}}_{\text{t}}=\;\text{c}0+{\text{c}}_{1}^{*}{\text{D}}_{\text{t}-1}+\text{c}2^{*}{}^{*}{\text{D}}_{\text{t}-1}+{\text{c}}_{3}^{*}{\text{D}}_{\text{t}-3}+\ldots +{\text{c}}_{\text{k}}^{*}{\text{D}}_{\text{t}-\text{k}}\\ \;+{\text{d}}_{1}{}^{*}{\text{S}}_{\text{t}-1}+{\text{d}}_{2}{}^{*}{\text{S}}_{\text{t}-2}+{\text{e}}_{2\text{t}} \end{array}$$

The interaction between population growth rate and per capita GDP is investigated using vector regression analysis. We conducted an ADF unit root test (pass) and residual diagnosis on the data and finally selected the VAR model with optional lag order of 3 for the causality test. The specific results are shown in Table 9.

**Table 9:** Granger causality test (at 5% test level).

**H0**	**F—Statistic**	**P**	**Conclusions**
Population growth rate–>GDP per capita	0.761	0.491	Accept
GDP per capita–>Population growth rate	5.091	0.014	Reject

It can be seen from Table 9 that in the long run, at the significance level of 5%, the population growth rate has no significant impact on the economic development level, while the economic development level has a significant impact on the population growth rate. Further, through the test of the co-integration relation between them, it can be known that there is a co-integration relation between them, and the co-integration equation is (the number in parentheses is t statistic value):


(12) $$\begin{array}{l} Co\text{ int }Eq1:GDP\;per\;capita=1711.36-39.14\\ \;\times Population\;growth\;rate\;(t=2.46) \end{array}$$

The results show that, in the short term, the population growth rate has no significant effect on the economic development level at the significance level of 5%, while the economic development level has a significant effect on the population growth rate. There is a significant negative correlation between population growth rate and economic development level in the long run. Thus, the hypotheses of H4a and H4b have also been proved.

## Conclusion

In this paper, based on the China agglomeration effect and the basis of the integration of development and also on 2008–2020 older triangle panel data of 41 cities of Anhui province, the Shanghai comprehensive index built high-quality development, using static panels fixed effect model, spatial econometric model, and the mediation effect model, as well as multidimensional examined population agglomeration of economic influence mechanism of the development of high quality. The study concluded as follows: (1) the population agglomeration of its influence on the development of the economy, high-quality “inverted U” type nonlinear characteristics, with a population agglomeration degree of ascension, the present quality of economic development at first, the influence of lowered later whether considering a spatial effect, the nonlinear relationship exists, and in the long triangle heterogeneity in different cities, in addition to the overseas. The other 40 prefecture-level cities did not reach the inflection point of the “inverted U” curve, that is, only Shanghai region has an overcrowding effect, which is not conducive to the high-quality development of the urban economy. (2) Population agglomeration can exert an urbanization effect to promote high-quality economic development. (3) The existence of fiscal decentralization significantly inhibits the promotion effect of population agglomeration on high-quality development; (4) Under the realistic basis of spatial convergence and spatial dependence, the improvement of neighboring economic development levels will also improve the local high-quality development levels. (5) In the short term, the population growth rate has no significant effect on the economic development level at the significance level of 5%, while the economic development level has a significant effect on the population growth rate. There is a significant negative correlation between population growth rate and economic development level in the long run.

## Data Availability Statement

The raw data supporting the conclusions of this article will be made available by the authors, without undue reservation.

## Author Contributions

YY: programming, writing, ideas, models, data, and typesetting. LL: writing, proofreading, data, and models. All authors contributed to the article and approved the submitted version.

## Funding

This work was funded by The National Social Science Fund of China (No. 21CRK010).

## Conflict of Interest

The authors declare that the research was conducted in the absence of any commercial or financial relationships that could be construed as a potential conflict of interest.

## Publisher's Note

All claims expressed in this article are solely those of the authors and do not necessarily represent those of their affiliated organizations, or those of the publisher, the editors and the reviewers. Any product that may be evaluated in this article, or claim that may be made by its manufacturer, is not guaranteed or endorsed by the publisher.
